# The importance of patient engagement when promoting age-inclusive research

**DOI:** 10.1186/s40900-025-00780-6

**Published:** 2025-08-22

**Authors:** Lydia E. Roberts, James O. Burton, Manish D. Sinha, Jonathan Barratt, Louise Oni

**Affiliations:** 1 Department of Cardiovascular Sciences, University of Leicester, Leicester, UK; 2 Department of Paediatrics, University Hospitals of Leicester NHS Trust, Leicester, UK; 3 John Walls Renal Unit, University Hospitals of Leicester NHS Trust, Leicester, UK; 4 Department of Paediatric Nephrology, Evelina London Children’s Hospital, London, UK; 5 Kings College London, London, UK; 6 Department of Women’s and Children’s Health, University of Liverpool, Institute in the Park Building, Alder Hey Children’s NHS Foundation Trust, Eaton Road, Liverpool, L12 2AP UK; 7 UCL Centre for Bladder and Kidney Health, London, UK; 8 Department of Paediatric Nephrology, Great Ormond Street Hospital for Children, London, UK

**Keywords:** Renal dialysis, Pediatrics, Patient participation, Research design

## Abstract

Children and young people continue to face many barriers to actively participating in clinical research, despite the same research questions being just as applicable to them as their adult counterparts. Exclusion of paediatric populations from drug trials can result in a lack of timely, equitable access to disease modifying medications. In other circumstances, there is the risk of extrapolating evidence from adult populations that may not meet the needs of a young population. In this brief report, we highlight the importance of directly involving children and young people when evaluating inclusion in research studies. This is illustrated by our service evaluation investigating current paediatric haemodialysis practices and opinions, and how these relate to a UK based clinical trial called the ‘NightLife study’ that is being conducted in adult haemodialysis centres. We discuss how age-inclusive approaches are aiming to change the paediatric clinical research landscape going forward, using research initiatives built on the principles of equity and inclusivity.

## Abstract (plain language summary)

Children and young people are often left out of clinical research, even when the same health questions apply to them as they do to adults. This can delay access to new treatments for young people or lead to adult research being used inappropriately to guide their care. In this article, we explain why it’s important to involve children and young people directly when deciding who should be included in research studies. We share findings from a survey looking at the current experiences and opinions of children receiving haemodialysis, and how these relate to a UK research study called the NightLife study, only open to adults. We show how efforts to make research more inclusive are helping to change how paediatric clinical trials are designed, with a focus on fairness and giving young people a voice.

## Introduction

Paediatricians are only too familiar with the practice of prescribing medications off license or relying on evidence extrapolated from clinical trials conducted exclusively in adult participants [1, 2]. These problems arise from a historical legacy where most clinical research trials excluded recruitment of, or simply failed to consider, the research question for children and young people who may experience the same disease [3]. Whilst there are unique ethical, moral, and legal considerations surrounding research in children [4, 5], due to the highly regulated environment, the current era means that by being denied the right to participate in research, children are potentially coming to harm by missing out on advances in health care outcomes.

The use of sodium-glucose transport protein 2 (SGLT2) inhibitors for the treatment of patients with chronic kidney disease (CKD) is a prime example of an emergent disparity in the standard care received by adult and paediatric patients. These drugs have been extensively tested in adult populations and have been shown to both slow the progression of CKD and reduce cardiovascular events [6, 7]. They represent a recent paradigm shift in the management of adults with CKD, providing a much-needed addition to the pharmacological toolkit to limit patient progression to kidney failure. As such, adult patients are now predicted to gain many years, even decades, free from dialysis due to these agents alone [8]. SGLT2 inhibitors however remain untested in a robust manner and unlicensed in children [9]. This is despite this population being at most risk of adverse outcomes including long-term social, emotional, and financial costs to the child, their family, society and healthcare systems in general, with irreplaceable years of childhood education potentially being lost to time on dialysis [10]. The use of SGLT2 inhibitors in adults has led to a new standard of care for adult patients, but unfortunately like many inequalities, exclusion of evidence generation for SGLT2 inhibitors in children will result in further exclusion from future CKD trials evaluating benefits of newer agents (versus standard of care).

## Scoping the feasibility of a paediatric trial – importance of patient engagement

Replicating studies in the paediatric population is not always as simple as just ensuring trials recruit patients of all ages from the outset or mirroring successful trials in adults in children and young people [4, 11]. An age-inclusive approach needs to consider the suitability of studies for the specific population. This has been exemplified following a recent service evaluation and patient engagement project designed to explore opportunities to adjust paediatric haemodialysis practices.

The NightLife study is a multicentre randomised controlled trial investigating whether overnight in-centre haemodialysis improves the quality of life of adult patients with kidney failure, compared to shorter (standard-of-care) daytime haemodialysis sessions (ISRCTN87042063) [12]. Over a 6-month period, participants in the intervention arm will receive 6–8 h of in-centre haemodialysis delivered overnight, 3 times per week, whereas participants in the standard-of-care arm will receive 3.5–5 h of in-centre haemodialysis, 3 times per week during the day. We sought to explore the opinions of children and young people currently receiving traditional day time hours haemodialysis to establish if replicating a similar trial in a paediatric population would be desired.

Following registration with the Alder Hey Clinical Audit Department (audit number 7148), online service evaluation questionnaires were developed and distributed to 13 paediatric haemodialysis units in the UK who represent the entire British Association of Paediatric Nephrology network. The patient/parent and staff questionnaires consisted of 17 and 22 core questions respectively. The questions had a mixture of response types including multiple choice options and free text answers, with additional optional opportunities to provide free text explanations for their responses. Questions explored current haemodialysis practices and opinions regarding preference for evening or overnight haemodialysis as alternative regimens. In total 24 responses were received from paediatric haemodialysis patients (2), parents (3) and paediatric haemodialysis staff (19) capturing feedback from 8 of the 13 (62%) paediatric haemodialysis units. All patients and families who provided their views were currently receiving daytime haemodialysis. Whilst 3/5 (60%) of patients/parents felt that evening dialysis would be beneficial for education, only 1/5 (20%) and 2/5 (40%) thought it would benefit their family life or hobbies respectively. In contrast, no patients/parents thought that overnight dialysis would be better for their family life, and only 2/5 (40%) thought it would be beneficial for their education or hobbies. The main reasons children and their families were opposed to consideration of evening or overnight haemodialysis included the importance of children sleeping in their own bed and maintaining their evenings free from dialysis to take part in activities they enjoy. Patients and their families also had several concerns regarding disrupted sleep during overnight dialysis and reduced out-of-hours staffing levels to manage any high-risk intradialytic complications that may occur in this vulnerable population. Concerns relating to limited out-of-hours staffing were also shared by most paediatric nurse respondents (16/19, 84%), with only 4/19 (21%) of staff willing to work night shifts to deliver overnight haemodialysis if needed as part of a study on different methods of in-centre haemodialysis.

Although this was only a small sample, clear and consistent themes emerged in respondents’ free text answers. The results highlighted that a trial in overnight haemodialysis in the paediatric setting would be at risk of not recruiting successfully. Increased social time provided by overnight dialysis may appeal to certain adult patients, however such benefits appear less applicable to children, with spending evenings and night-time at home holding significant importance for their social and emotional development. Maximising time in education is crucial for children on dialysis, who miss out on reaching their potential, however our findings demonstrate that alternative ways to achieve education whilst on dialysis must be explored. As well as suggesting overnight haemodialysis may be inconvenient for many paediatric patients, our service evaluation flagged valid concerns regarding the safety of performing paediatric haemodialysis out of hours. It is well documented that lower nursing staffing levels are associated with increased risks of patient morbidity and mortality [13–15]. Although the patient-to-staffing ratios remain the same for nocturnal dialysis, due to the smaller number of people receiving dialysis overnight, the absolute number of staff in attendance is lower. In addition, since overnight in-centre dialysis is delivered out-of-hours, medical cover would be provided by on-call staff only. Although there have been no reported safety concerns in the adult haemodialysis population, children are known to be more prone to several haemodialysis-related complications than adults including intradialytic hypotension [16, 17] and thus performing haemodialysis overnight may carry an unacceptably high perceived risk in paediatric patients.

## Conclusions

This work highlights the importance of ensuring that children are considered from the outset when designing age-inclusive research during this unique period in life. Excluding paediatric patients from studies merely for simplicity or convenience is hard to justify, however, in some cases it may be that there are genuine reasons that a trial in adults would not be relevant or appropriate for extension into paediatric populations. Our results suggest that expanding the NightLife trial to recruit children could have wasted additional time and precious resources on an extended trial design. The use of specific patient and public involvement (PPI) in the paediatric setting is required to ensure research aims and objectives are applicable and meaningful to those with lived experience of the disease in question.

Further work in this area includes the creation of a speciality specific children’s kidney advisory board that will be involved in informing the suitability of other research hypotheses, project design, and selection during the grant award process. Paediatric nephrology has a unique opportunity to lead a radical change to champion equitable and age-inclusive research in the UK and beyond. The recently launched £10.4 M LifeArc-Kidney Research UK Centre for Rare Kidney Diseases has been created with children at the centre to transform rare kidney disease research, an entity that encompasses all childhood onset kidney disease [18]. By uniting clinicians, scientists, patients and charities to put children first, this ambitious collaborative project will work to ensure children and young people equitably benefit from advances in medical knowledge and practice going forward.

## Data Availability

The datasets used and/or analysed during the current study are available from the corresponding author on reasonable request.

## Abbreviations

CKD: Chronic kidney disease
PPI: Patient and public involvement
SGLT2: Sodium–glucose transport protein 2
